# The Ideal Body: A Social Construct? Reflections on Body Pressure and Body Ideal Among Students in Upper Secondary School

**DOI:** 10.3389/fspor.2021.727502

**Published:** 2021-10-11

**Authors:** Ove Olsen Sæle, Ida Kathrine Sæther, Nina Grieg Viig

**Affiliations:** ^1^Faculty of Education, Arts and Sports, Western Norway University of Applied Sciences, Bergen, Norway; ^2^Ålesund High School, Ålesund, Norway

**Keywords:** body ideal, body pressure, Foucault, social media, young people, panopticon, synopticon, omniopticon

## Abstract

Several studies show that young people today are negatively impacted by body image ideals in social media. We studies young people's reflections on body image and body pressure. More precisely: How does a selected group of third-year upper secondary school students understand their body images and body pressures through social media? Eight third-year students, four of each gender, were interviewed from two upper secondary schools in a medium-sized city in Norway. An interesting find was that body pressure was not experienced solely as one-sided pressure exerted externally by media sources, but that they also personally influenced others through their own active use of social media channels like Facebook and Instagram. They reported having experienced body pressure in their own lives and in their immediate environment, and that both genders are affected. The study builds on sociocultural body theory based on Foucault's ideas and work, but also uses more recent media theory for the analysis and discussion. A BOPS model developed by the researchers was used for the operational parameters that is centered around the concepts of panopticon, synopticon and omniopticon.

## Introduction

According to Giddens (1997), we live in a secular age in which individuals are responsible for defining their own body, free to (re)shape the body “in our image” or in line with the body ideals of the time. Young people seem to be particularly influenced by this body culture, a topic that has been the focus of several Norwegian TV shows, such as *Sykt Perfekt* (Insanely perfect), *Skam* (Shame), *FML* (Fuck My Life), and *Jeg Mot Deg* (I Against You)^1^. This article presents findings from a study that examined in detail how upper secondary school pupils reflect on the topics of the body and body pressure. We first present previous research within body pressure, then our theoretical framework and a method section and finally the findings and discussion.

## Previous Research Within Body Pressure

Previous research addresses whether Norwegian children and young people today are exposed to body and appearance pressure from social media, where the ideal body is portrayed (Øgård-Repål et al., 2017; Østvold, 2017)^2^. According to Holmqvist (2013), young people exist in a distinctly idealized and standardized body culture, where the idea of external physical beauty is presented as ideal, created and maintained by the mass media, advertising and the fashion industry. In the national *Ungdata* (Youth Data) survey for 2019, several interesting figures emerged (Bakken, 2019)^3^. On the question of whether young people experience pressure to look good or to have a nice body (ibid., p. 72), as many as 70% answered in the affirmative (31% a little pressure, 17% some pressure, 11% quite a lot of pressure, and 12% very much pressure). More girls (35%) than boys (10%) responded in the affirmative regarding pressure to look good. In addition, 9% of boys and 11% of girls have experienced somebody posting hurtful photos or videos of them online or via mobile phones. Bratland-Sanda and Sundgot-Borgen (2011) also pointed out that there are more girls at a normal weight than boys who want to reduce their body weight, while there are more boys at a normal weight who want to increase it.

In parallel with this assumption of the existence of a contemporary body idealization, some international studies indicate that dissatisfaction with one's own body is not uncommon, among young people of both sexes, a tendency that can help reinforce an assumed body pressure (Holsen et al., 2012; Carey et al., 2014; Tatangelo and Ricciardelli, 2017). Smolak and Stein (2010) also discovered that boys who built muscular bodies were closely related to the fact that they compared themselves to muscular ideal bodies in the media.

However, it is important to note that most children show great satisfaction with their own bodies (McCabe and Ricciardelli, 2004). The variables that increase the risk of body dissatisfaction appear to be the same for both sexes, while girls tend to be more dissatisfied with their own bodies than boys (Knauss et al., 2008; Holsen et al., 2012; Tiggemann and Slater, 2013; Holland and Tiggemann, 2016,^4^). It has also been shown that body image dissatisfaction has been more prevalent among female adolescents compared to males and being a woman has been positively associated with higher problematic use of social media among adolescent students (Gestsdottir et al., 2018; Kircaburun et al., 2018). Slater and Tiggemann's (2014) study of teenage boys showed that different media genres seem to trigger boys' desire to become thinner or more muscular to varying degrees; sports and entertainment magazines could trigger a desire for bigger muscles, whereas reality TV shows could trigger a desire to become thinner.

However, this conclusion may be attributable to the fact that there is more information available regarding the factors and developmental trends in body image in girls than boys (Smolak, 2004), or because studies have mostly focused on issues relevant to women (McCabe and Ricciardelli, 2004). It is also the case that thin female bodies are more often portrayed in the advertising and media industries that muscular male ideals (Tiggemann and Slater, 2013, 2014). It also does not appear that this gender difference evens out with advancing age (Bratland-Sanda and Sundgot-Borgen, 2011).

It seems to be a widespread phenomenon that adults and adolescents are exposed to idealized body images in social media such as Facebook and Instagram (Tiggemann et al., 2020). There are for example several studies that show a close connection between Facebook usage and young women's body image concerns (Tiggemann and Slater, 2013, 2014; Mabe et al., 2014; Cohen et al., 2017). This also applies to men (Mabe et al., 2014) and girls and boys (Ringrose, 2011; Flynn, 2016; Trekels and Eggermont, 2017; Trekels et al., 2018).

An overview study showed increased awareness of one's own body in connection with exposure to attractive Facebook profiles and using social media in general (Holland and Tiggemann, 2016). Studies shows that participants who spent time on Facebook reported being in a more negative mood than those who spent time on the control website (Mabe et al., 2014; Sagioglou and Greitemeyer, 2014; Fardouly et al., 2015).

The same tendencies apply when it comes to Instagram, where you also can post the best and sometimes manipulated self-portraits (Tiggemann et al., 2018, 2020; Brathwaite and DeAndrea, 2021). An overview study indicates that there have been smaller studies on the use of Instagram for young people, and from the existing studies it is concluded that online selfies have a negative impact on adolescents' well-being and body confidence (McLean et al., 2019).

Some Norwegian studies also show that young people are affected by the media and advertising industry's displays of body ideals and the resulting body pressure (Eriksen et al., 2017; Østvold, 2017, Øgård-Repål et al., 2017). Østvold discusses the presentation of narrow beauty ideals in advertisements, the use of comparisons and a tendency to normalize cosmetic surgery.

Holsen et al.'s (2012) study showed that adolescents with a good relationship with their parents and friends seem to be more satisfied than those with a poor relationship with their parents and friends. Grossbard et al. (2011) point out that one's perceptions about the body are influenced by expectations set by the opposite sex, and these perceptions are often distorted. This finding emerged from their quantitative study of gender differences associated with body dissatisfaction and misconceptions of norms of thinness and muscularity. Furthermore, Sand et al. (2011) conducted a quantitative study of Norwegian adolescents' ability to estimate the size of different bodies. The results showed, among other things, that perceived body pressure and body dissatisfaction can also be due to a person's psychological characteristics, such as a lack of self-esteem or self-confidence, which corresponds to some of the mentioned international studies.

As shown in both Norwegian and international research, there are several studies that have examined which sources young people are influenced by in terms of body ideals, especially from social media platforms. However, few studies have used interviews to elicit the informants' authentic “voices” in this field. In our research, we examined in detail how upper secondary school pupils reflected on the topics of the body and body pressure. We asked: How does a selected group of third-year upper secondary school students understand their body images and body pressures through social media?

In the discussion, terms such as *body pressure, body image* and *body ideals* are used. Body pressure is defined by the Norwegian Media Authority (Medietilsynet., 2018) as: “Pressure that is problematic to deal with, and which makes the person who experiences such pressure have a negative body image, feel dissatisfied with their body, or be ashamed or embarrassed about the way they perceive themselves to look” (p. 3). They point out that there is a difference between commercial body pressure, from actors such as social media, and body pressure that comes from friends and peers. Body image includes a person's thoughts, feelings and perceptions of his or her body (Grogan, 2008), largely based on the body's shape and size Grogan also links body image to whether one considers their body to be aesthetically and sexually attractive. In other words, one's body image is linked to the body ideal that one attempts to pursue. One's body ideal will then apply to “the body type that is regarded as most suitable and attractive for a person, considering one's age, gender, build and culture (Pam, 2013).

While there is significant research on young people's body image concerns, they do not typically consider how social control impacts these issues. In this article, we bring in Michel Foucault's sociocultural theory of power, discipline and control to further understand body pressures stemming from social media.

## Theoretical Framework

The theoretical basis of this article is based on the French philosopher Michel Foucault (1926-1984) and his sociocultural interpretation of the body. His theory has been applied to various disciplines of research on the media (Duncan, 1994; Holsen et al., 2001; Edy and Snidow, 2011; Ringrose, 2011; Ringdal, 2013; Allain and Marshall, 2017), sport (Clark and Markula, 2017), physical education (Augestad, 2003; Azzarito, 2009) and fitness culture (Markula, 1995; Allain and Marshall, 2017). Foucault talks about the disciplined body. In his classic work, Discipline and Punish (Foucault, 1977), he perceives the 18th century as the beginning of today's strict project of body discipline. One of his main theses is the claim that the body will, in any cultural context, be subject to some form of control and discipline.

Foucault perceives state influence and regulation of the body as a strategy of state power and calls it “political anatomy” (p. 127); a political strategy concerning the body, which “teaches how to attain the body of others” (p. 127), a body that “can be subdued, used, transformed and perfected” (p. 126). The conceptualization of anatomo-political power on the human body is expressed in the form of various disciplinary techniques that administer and regulate the individual citizen's body and contribute to us in society producing obedient and what Foucault calls docile bodies (Foucault, 1977). His concept must not be perceived as a fixed or stationary concept of power owned or exercised by authorities or individuals alone, but rather constitutes as a dynamic and relational form of power internalized by the individual citizen. Foucault (1972) and Lemke (2019) points out that power cannot be perceived as a static quantity belonging to specific individuals, institutions or states, but it must always be interpreted in the light of the social context in which it operates.

Central to Foucault's understanding of power as a relational, social phenomenon is the gaze. The gaze which he calls “the perfect eye” that notices everything, to which all eyes are directed (Foucault, 1977, p. 158). He uses the *panopticon*^5^, as a model for this power apparatus. It was built so that the prison guard could see all the prisoners from his watchtower, without them being able to see him. The panoptic system enables “the few” to watch and manipulate “the many.” Through the perpetual gaze, prisoners were disciplined to monitor their own behavior in a way that make them compliant (Foucault, 1977; Shilling, 2003; Cole et al., 2004; Markula and Pringle, 2007). Foucault use the term governmentality, which emphasized that the panoptic arrangement (and external gaze) is not a form of “external” or “one-side” mechanisms of power from the authorities, but must be seen as a subtle and diffuse power that has been internalized in the individual and which acts as a form of self-monitoring self-government (Thualagant, 2011).

Duncan (1994) refers to Foucault and “the panoptic gaze” to emphasize that the panopticon prison is an expression of a metaphorical gaze in which the individuals internalize the gaze. With reference to Bartky (1990, p.74), she points out that such a panoptic gaze is an invisible and dynamic disciplinary mechanism and power that refers to a body that is “everywhere and nowhere, is everyone and yet no one in particular.” Duncan uses such a panoptic gaze in her analysis of fitness magazines, where it is illustrated in form of ideal bodies and alluring body rhetoric. She points out that such a panoptic gaze (on the body) can be expressed on different levels and in different forms in social media, and lead to bodily dissatisfaction especially in women.

Some believe that we are facing even more power strategies today and that modern society constitutes a disciplinary society, not least in view of the rapidly expanding media culture of our time (Markula and Pringle, 2007; Slater and Tiggemann, 2014; Holland and Tiggemann, 2016; Cohen et al., 2017; Tiggemann et al., 2020). The term *synopticon*^6^ was later introduced as a theory that takes into account the today's media society where “many see few.” Here, a form of human consciousness is imagined whereby the individual in the consumer society is uncritically influenced by social media and is not able to distinguish the real from the false (Mathiesen, 1997, 2004; Azzarito, 2009). Mathiesen (1997) argues that the panoptic and synoptic structures combine to constitute crucial control functions in modern society.

To understand the media society of our time, there are also those who refer to the term *omniopticon*^7^ (Doyle, 2011; Jurgenson, 2011; Marwick, 2012). It is a monitoring mechanism with a horizontal power structure whereby “the many see the many.” In the omniopticon, one is not only a passive *consumer*, but also a producer of content, which makes the individual a *prosumer* within the realm of social media (Jurgenson, 2011). Vanden et al. (2018) refer to today's digital media as an example of a panoptic *and* an omnioptic tool of power. Marwick (2012) connects the omniopticon with the concept of *social surveillance*. She presents various forms of social power and explains how they are expressed through social media technologies. On digital platforms such as Facebook, Instagram and Snapchat, one shares personal information (such as photos of the body) in order to be seen by people and receive “likes” (Tiggemann et al., 2018, 2020). Here, power is shared by those who participate online, because they can mutually influence each other. This is in line with Foucault's dynamic concept of power, which Marwick also uses in his analysis.

**Figure 1:**
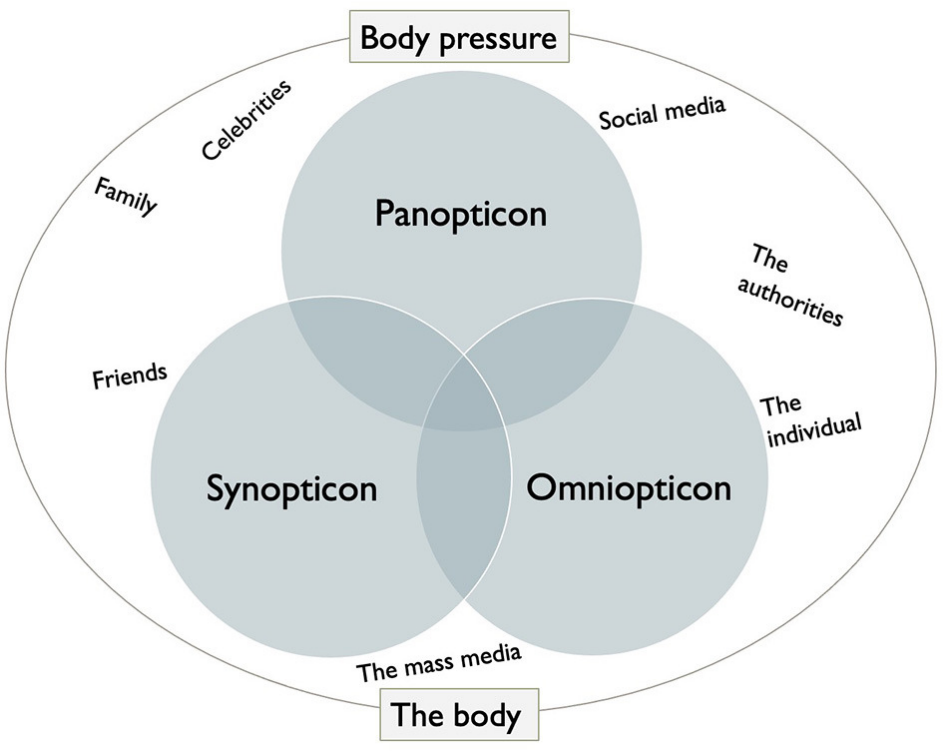
The BOPS model. Various sources of influence regarding body image and body pressure among young people.

The BOPS model (Figure 1 below), an abbreviation for “body and body pressure, omniopticon, panopticon and synopticon,” summarizes the theoretical concept, and serves as a structural framework for the discussion. The model illustrates the various power strategies and power structures. Reference is made here to the various sources of influence that the informants reflect on, related to the body and body pressure. We would argue that our model of expanding a panopticon power strategy to include panoptic and omnioptic power strategies, with the development of new social media platforms, corresponds with Foucault's concept of a dynamic power strategy. His basic thinking that the panoptic power strategy takes place from above on the part of the authorities and at the same time is integrated into the individual in a subtle and invisible way, illustrates that it is a dynamic power strategy that can manifest itself as both a vertical and horizontal power structure. This means that the public can also actively use social media in their communication about the body. The open question is whether the audience on new media platforms such as Facebook and Instagram relay the same body message that they have received from the media and advertising industry.

## Materials and Methods

This article builds on an interview study conducted with upper secondary school pupils.

### Sample

A strategic sample was selected, consisting of eight 18-year-old third-year students at two different upper secondary schools in a medium-sized city in Møre og Romsdal county, Norway. Prior to the recruitment process, equal gender distribution, third-year upper secondary students, two different lines of study, voluntary participation and written informed consent were defined as inclusion criteria. The informants were thereupon chosen from two different lines of study: general studies and elite sports. The gender distribution from each school was equal: two boys and two girls. The two lines of study were chosen with a view to having the most representative sample possible, consisting of those more active in sports and those who are more passive (i.e., general studies). Nevertheless, it emerged that seven of the eight informants participated in sports in their spare time. Third-year upper secondary pupils were chosen because they are young people who are hopefully able to provide reflective answers. The equal gender distribution was important given the fact that gender is often a key variable in studies based around young people and their bodies.

### Data Collection

Contact with schools took place via school leaders and then through individual teachers who helped with the recruitment of students. Interviews were conducted, which is an appropriate methodological approach for in-depth studies whereby the purpose is to reveal informants' opinions, attitudes and experiences (Tjora, 2017). Interviews and later analysis of the material is also an appropriate method for discourse studies, because it provides insight into how people construct their reality through the ways in which they express themselves (Thagaard, 2018).

A semi-structured interview was chosen to record the informants' views on the body. Here, a formulated interview guide was used, divided into four topics (social media, own relationship with the body, body ideals and body pressure). The interview guide was developed with open-ended questions, thus providing guidance for the conversation while at the same time allowing for more open dialogue, with the possibility of follow-up questions (Kvale and Brinkmann, 2015). Studies show that informants often fail to express themselves in the first person when discussing the body, or that they distance themselves from topics that deal with body pressure (Lauritzen, 2016; Eriksen et al., 2017). Due to the sensitivity of the topic, the interviews were conducted individually, as opposed to in a focus group.

The interviews were conducted in the autumn of 2018 by one of the authors, lasted for approximately 20 to 45 min and were audio recorded. Despite improvements of the formulations in the interview guide following the conducted pilot interview, a few questions appeared unclear to some of the informants. One of the interviews was interrupted on account of the need to change rooms, which led to the absence of certain questions. However, the rest of the interviews were conducted in their entirety.

### Data Analysis

The audio recordings of the interviews were transcribed and coded in main and subcategories in the program NVivo 10. The first step in the analysis was to conduct an empirical coding, where the point is for the codes to be as close as possible to the informants' utterances, dealing with the minutiae of the material (Tjora, 2017). All utterances were given individual codes. The coding was carried out to ensure that the statements were represented in their entirety. The codes were then classified into relevant main categories with subcategories (Ringdal, 2013).

The main categories were primarily based on the interview guide's thematization, but the topics “own relationship with the body” and “body ideals” were merged into “body.” Some of the subcategories, such as “what makes this an ideal body?” was created based on questions in the interview guide. Other subcategories, for instance “the choice to be influenced” and “body as an object” were created based on the content of the codes.

The interviews were based on how the informants assign meaning to their experiences through the ways in which they express themselves. The focus was therefore on identifying the views that were reflected in the informants' understanding of the body and body pressure and on analyzing their statements considering theories and previous research.

### Ethical Considerations

The informants' identities were hidden through anonymization, and their pseudonyms were omitted from all paraphrasing. One can unconsciously experience a “biased viewpoint effect,” a possible error source whereby the researcher emphasizes a specific perspective as being representative at the cost of other perspectives (Ringdal, 2013), which can threaten the reliability of the research. We understand that interviewing young people in relation to sensitive topics such as body pressure, requires a relationship of trust in the interview situation that we do not know for sure has been achieved. Talking about body image and body-looking pressure can be perceived as sensitive and potentially lead to more destructive emotions related to body image. This was considered in the interview section in that it was stated that the informant did not need to provide detailed personal information of a more private, sensitive nature. In addition, based on the sensitive topic of the interviews, the information form included contact information for different school nurses. None of the informants received any rewards from taking part in the study.

The Norwegian Centre for Research Data (NSD) has assessed the study and is in line with privacy regulations. In accordance with NSD's guidelines, all the informants provided a written informed consent. The information form contained details regarding the purpose of the study, the interview situation, the opportunity to withdraw from participating, privacy, the use of pseudonyms to ensure anonymity and that audio recordings and any other personal information would be deleted at the end of June 2019.

## Results and Discussion

When presenting and discussing the results of this study, an attempt has been made to give a presentation of all the different perspectives that emerged through the interviews. On some occasions, several quotes with common content have been collected into a common voice, while when one or more informants have stood out from the crowd, direct quotation or paraphrasing is used.

### Synopticon—The Many Seeing the Few

The synoptic power perspective, or Foucault's thinking about the possibilities for institutions to manipulate the thoughts and practices around the body for the majority (Foucault, 1977; Mathiesen, 1997, 2004), seems to be confirmed by several of the informants in our study.

Most of the girls in the study give a more concrete description of the make-up of the ideal body, while the boys relate it to ideal-typical formulations such as “the perfect body” and “the dream body”:

I think it's like the ideal body becomes a part of what you want. […] If you want to look well-trained and stuff, then you have to have that kind of lifestyle. And if that lifestyle is something you have to work hard for, then I wouldn't say it's an ideal body because it affects your lifestyle. As long as you are fine with your body and can do the things you want, there's no real answer as to what the ideal body is. (Lars)

Muscles are a key theme represented in the boys' perceptions of the ideal body: “It's kind of like they should be as big as possible, with the biggest muscles and stuff like that” (Andrea). Several of the boys refer to the importance of having a trained, flat stomach and a six-pack, depictions that correspond to the notion of a well-proportioned muscular body as being the male body ideal (Markula and Pringle, 2007). It is also in line with Bratland-Sanda and Sundgot-Borgen's (2011) study of Norwegian teenagers, where a desire for muscles and physical activity were prominent patterns. It seems that the muscular body ideal becomes synonymous with a disciplined, trained body (Smolak and Stein, 2010).

Some of the informants describe a body ideal related to being well-trained in order to perform in areas of life where exercise and physical activity are key. This may indicate that they have a clear perception of what the media and society view as the ideal male body, but that they themselves challenge this ideal by focusing on the body's performance rather than how fit the body looks (Rønbeck, 2012). One informant states that most boys would agree with his description of the ideal body, a statement that may reflect a media discourse around the body of which he himself has become a part (Thualagant, 2011).

Our study demonstrates that there is a mutual influence between specific perceptions and practices related to the body. It appears that body ideals are closely linked to exercise and training, which corresponds with Slater and Tiggemann's (2014) study, showing that teenage boys referred to the body images they had seen in various sports magazines. The BOPS model is seen here through the synoptic power dynamic, which indicates that the ideal stems from impressions that the individual receives from external sources.

We also found that the girls' body ideal appears to be more complex than the boys'. All of the girls describe the ideal body for girls as thin, with large buttocks and breasts. Some also mention things related to the stomach, waist, legs, arms, face, and hair. Sonja says:

It's perhaps not a very healthy image of an ideal body, but I tend think of tall, thin models. Maybe not the thinnest ones, but one should still be tall, thin, quite well-toned, and have a nice face. Everything should be so perfect. And even though one should be thin, one should still have boobs and a bum.

This seems to correspond with Markula's (1995) study of the female body image in aerobics. She discovered through ethnographic fieldwork, interviews, and media analysis, that the media ideal is a contradiction: firm but shapely, fit but sexy, strong but thin.

One of the girls reflects on body ideals as something current, defined, and self-constructed, closely linked to media presentation:

I think that in a way it is something you create in your own head, and you are so pressured to think that there is a body ideal that you get a bit carried away with the fact that there is so much written about it in the news and such places, that there is this great ideal of being slim, thin, tall and stuff. (Andrea)

The informants' descriptions of the ideal girl's body being thin agree with Bratland-Sanda and Sundgot-Borgen's (2011) findings about a negative linear relationship between girls' satisfaction with their own bodies and BMI. Markula and Pringle (2007) estimate that only five percent of all women are born with the right genetics to make the current idea of the perfect body attainable.

The girls seem to be divided on whether they make a distinction between the general body ideal in society and their own personal body ideal. One states that her idea of an ideal body is a “normal” body that is not too big or too thin, a finding that is consistent with Grossbard et al.'s (2011) study, which showed that the informants' own body ideals consisted of larger bodies than they perceived other women to view as ideal. Another says that the ideal body is “the one that works best and makes me feel comfortable in my own body” (Andrea).

Describing the ideal body as functional shows that the idea of the perfect body is not only based on aesthetics, demonstrated by some of the male informants who were concerned with the body's performance. The fact that some girls distinguish between a general body ideal and their own may indicate that they, like some of the boys, are aware of the media's and society's discourse about the ideal body and that they wish to challenge it (Rønbeck, 2012). Markula (1995) points out that several of the female aerobicizers in her study were influenced by the body ideals in exercise magazines (text and pictures), but at the same time many were also skeptical of them. She concludes that (p. 450): “Woman's relationship with the body ideal is contradictory. This awareness, nevertheless, demonstrates that women have not internalized the panoptic [and synoptic] power arrangement entirely.”

A synoptic power influence seems to be evident in the informants' descriptions of how celebrities and other role models influence people, which may contribute to recipients not being able to distinguish the real from the false (Mathiesen, 1997, 2004; Azzarito, 2009). Nevertheless, we also see examples of informants being critical of this influence (Mathiesen, 1997, 2004; Azzarito, 2009).

Most of the informants, regardless of gender, associate the media's body ideals with “what is viewed as nice or what looks best,” and also with a person who is dedicated and structured: “It reflects a person being active and healthy, and people connect that with positive things” (Lars). Here, the ideal body is associated with success (Markula and Pringle, 2007; Grogan, 2008).

### Panopticon—The Few Seeing the Many

It may seem that the synoptic perspective is that which is most expressed among the informants, as they refer often to body exposure of models in magazines and advertisements, as well as celebrities on TV and on social media. It can be seen as an expression of the mass media's body-disciplinary effect where “the many see the few” (Mathiesen, 1997, 2004; Azzarito, 2009). However, one informant offers a somewhat nuanced picture and refers to both “health and training-related” and “body-fixated” perspectives (Ole):

On the one hand, you have the health benefits of having a well-functioning body, you can lift heavier weight, avoid getting tired quickly and so on. So this comes from research and also having it drummed into you from a young age, that it's important to exercise, eat healthy, stuff like that. […] But when it comes to appearance, it's social media that has the greatest impact.

Although it is not explicitly stated, it is reasonable to assume that the informant is referring to recommendations made by various health authorities, which may be reminiscent of Foucault's presentation of a panoptical hierarchical power strategy. Through state health campaigns, the individual is molded to think in a certain way, which contributes to a specific set of health and wellness practices (Augestad, 2005).

### Omniopticon—The Many Seeing the Many

One informant mentions young people's discussions of body ideals as a source of influence. This can be reminiscent of an omnioptic influence, where influence on body issues goes both ways. This can be in the form of structured conversations, offhand remarks or other kinds of comments that specifically refer to the body.

The informant refers to personal social media profiles, on Facebook, Instagram, blogs etc., where it is possible to respond to images depicting the body. We have previously referred to studies that show that both young people and adults' comment and post pictures of themselves on social media to get confirmation (see Fardouly et al., 2015; Flynn, 2016; Trekels et al., 2018; Tiggemann et al., 2020).

Here, the purpose is “to be seen,” but also to receive likes and other feedback. This contrasts with other forms of media, such as advertising and fashion magazines, where an opportunity to provide instant feedback is not available and where the purpose is to sell a product. In this instance, a response to the image is expected to be shared with the rest of the digital sphere, clearly marking a link between visibility and power (Markula and Pringle, 2007). If only the lightly-dressed bodies which closely resemble “the ideal body” are published on personal social media profiles, these bodies will appear to be the norm. This will contribute to those posting pictures of themselves becoming objects of a normalizing gaze, with all the subsequent consequences (comments), with the ideal body being used as a point of reference (Markula, 1995; Markula and Pringle, 2007). Comments may lead to a practice of sharing which, when assessed, distinguishes the normal from the abnormal (Cole et al., 2004).

From an omnioptic perspective, where “the many see the many” (Jurgenson, 2011), it is conceivable that the body ideal is supported by the social surveillance normalized by social media (Marwick, 2012). At the same time, the informants assume the role of *prosumers* in that they are producers of the content (Jurgenson, 2011). The posting criteria may indicate that what they share is regulated by their perceptions of the recipients (Marwick, 2012). Although social surveillance is considered mutual, it is nevertheless conceivable that it facilitates hierarchical observation (Foucault, 1977) through the individual's search for confirmation in the form of “likes.” Normalizing sanctions appear through individuals striving for likes. It seems that the more one receives, the closer they are to the ideal body. If the individual views the number of likes as a status symbol, it can lead to a desire to be viewed as normal (Markula and Pringle, 2007). Through their search for confirmation through likes, young people choose to share their best sides with one another through social media, which can create an unrealistic view of reality. Even if they assume that they are acting of their own free will, it is not unrealistic to think that an unconscious external influence may be at play (Hultqvist, 2011), from role models and other significant figures that they follow on social media.

### The Key Role of Social Media

Most informants say that social media has been an important contributor to the increase in body pressure. One informant explains this by pointing out that celebrities and other role models, through social media, have become more accessible than they were before. Another explains it as a general phenomenon; people perceive social media as creating pressure about needing to have the nicest clothes, be the best and look the best. The informant is also critical to the body exposure in the media to people who are already quite insecure about themselves, although she acknowledges that one can choose who one follows on social media.

The idea that some of that which is aimed at young people on social media concerns the body, and that social media is of great importance to young people, seems to support previous conclusions (Østvold, 2017), and that if one is exposed to body ideals on social media, it can contribute to one's own dissatisfaction with the body (Cohen et al., 2017; Tatangelo and Ricciardelli, 2017).

In order to create the best possible presentation of oneself on social media, some people choose to edit and “touch up” images. The informants say that it is mainly girls, or parts of the media who target girls, who edit pictures. None of the males know if the profiles they follow are edited, while most of the girls are aware of the fact: “There are so many apps you can do everything on, so you can never really be sure” (Maja). Many believe it is a pity that people resort to this type of editing, because it helps to maintain an unrealistic body image (Manago et al., 2008).

One male informant states: “I don't want girls to be skinny, but I feel that it's what they want. […] Girls want to be as thin as possible, but I don't feel that's what boys want” (Erik). This seems to support Grossbard et al.'s (Grossbard et al., 2011) findings which suggest that women distort what they believe to be men's preferences for female bodies. Informants also mention that, when people post pictures online of well-trained bodies, and others of overweight bodies, and only the former receives positive comments, it can contribute to increased body pressure and support the notion that only a specific body ideal is desirable. Ole states:

If you see that a well-trained person posts a picture of themselves, you will often see comments like “you're so nice” and the rest, but if, for example, an overweight person posts a picture, then they are described as “brave.” The person is seen as brave because they are far from ideal, but they are still comfortable enough to post the picture.

The comments that are attached to the various images of the body found on social media can be read by anybody who has access to the image and comment section. The holder of power and influence in this form of communication is not therefore limited to the sender and the recipient but can be viewed as a ubiquitous element of power and influence within a society (Thualagant, 2011). In this case, the social media platform functions as a synopticon where the many see the few (Mathiesen, 1997, 2004; Grabe et al., 2008; Azzarito, 2009; Want, 2009; Holmqvist, 2013; Øgård-Repål et al., 2017; Østvold, 2017).

Most of the informants state that social media plays a big role in the lives of young people in modern society and that it takes up a lot of their time. One informant justifies this by pointing out that all young people have access to social media through their mobile phones. However, there is some variation in how the informants define the role they believe social media has. Erik says: “There are many people who use Instagram a lot, and they post pictures of themselves where they look good in order to show off.” In relation to this, several of the informants believe that girls post more photos on Instagram than boys. Some studies indicate that the reason given for this is that girls are more concerned with what other people think and, to a greater extent than boys, need the extra confirmation (Holsen et al., 2012; Cohen et al., 2017).

Some of the informants point out that social media is used to have conversations, get to know people or plan activities with friends. Others believe that social media has an influence on young people by being the last word in the way that people should look. Most informants only follow their friends, family, and acquaintances on social media, while some also follow celebrities.

All the informants report posting photos, but to varying degrees. They describe various criteria for posting, such as the photo being nice enough to be posted on Instagram. Some say that a photo is nice if it depicts a special occasion or a sunset, while others associate nice photos with the physical appearance of the body. Maja explains: “You have to look pretty good […] Maybe a sweet sparkle in the eye and a nice smile. You don't post an ugly picture—you want people to see you at your best.” One of the boys says that he recognizes that he gets more “likes” on pictures where he looks especially good. One of the girls justifies posting the best version of oneself on social media by pointing out that one does not want to be vulnerable by posting their “bad sides.” This reflects previous findings which showed that both youths and adults post the best, and often manipulated, self-photos on Facebook and Instagram, to get attention in the form of likes (see for example Carey et al., 2014; Tatangelo and Ricciardelli, 2017; Tiggemann and Slater, 2017; Tiggemann et al., 2020).

### Synoptic, Omnioptic Panoptic Power and Body Image

Most of the male informants feel that their social environments largely consist of people with a good relationship with their own body. Ole says: “People don't always open up about things like that, so I don't know for sure. But nobody in the boys' changing room has an issue with being exposed.” The fact that Ole emphasizes their reluctance to open up about their relationship with their body could indicate that many boys experience discomfort about expressing dissatisfaction with their own appearance (Smolak and Stein, 2010). When boys make negative comments about their own bodies, it is often presented as humor, in that they “throw out silly comments,” which may serve the purpose of concealing the truth, namely that they are influenced by the idea of the “perfect body.”

The girls talk about different experiences related to the ways in which their social environments have shaped their body image. Several described how their mothers have always told them that they are nice the way they are. Maja says: “I have learned to love my body the way it is and to not be so affected.” However, another says that there is a lot of discussion of the body in her family, which has contributed to her also focusing a great deal on her body. She has a mother with a negative view of her own body, and she thinks it may have spread to her.

Girls' body images also seem to be influenced by peer groups, which corresponds to other studies (Holsen et al., 2012; Fardouly et al., 2015). One of the girls explains that, in the 10th grade, she went from training to develop her skills to training to look better:

I had never thought about whether the body should look like this or that, but then suddenly there was a big focus on bum, boobs, and waist. Suddenly, you had to look a specific way, and it was something I'd never thought of before. […] I kind of noticed it because others were talking about it and guys started commented on it, without it being meant badly. (Sonja)

She emphasizes that other people's views of her body mean a great deal to her and that, even though she receives positive comments about her body from others who are bigger than her, these comments have contributed to her looking for faults in herself. Another girl states that there was a lot of talk about the body as early as primary school, and she recalls a specific episode (Åsa): “I remember that I had some friends round at home, and I think I tried on some clothes. Then I got comments about looking nice as long as I sucked my stomach in a little. […] That was probably in sixth grade I think.” She says that many of her friends have a healthier relationship with the body than she has, but there are still some who talk about having to lose four kilos if they gain two. Another talks about her group of friends within which everyone is comfortable in their own bodies, and the body “is not a big deal.” She reflects that this has probably helped her to view her own body in a positive light and that she and her friends build one another up with these healthy attitudes. This corresponds to studies which show that the school context is important as it fosters observation and comparison among classmates (Mueller et al., 2010).

If one examines the discourse on the importance of the social environment for one's own body image in light of the BOPS model, it seems to support both a panoptic and omnioptic power relationship where the individual, family and friends are all relevant influencers and actors (Holsen et al., 2001). Normalizing sanctions also appear to come from family and friends, in that one receives assurances that they are “nice the way you are.”

In the context of this study, being satisfied with one's own body can be categorized as the norm. The fact that the girls generally have a positive view of their own body may indicate that they have become self-regulating, without strong external influences (Foucault, 1977). In terms of the informants who experience friends and family being a negative influence, it may be that others' negative views and focus on the body are experienced as an eternal, authoritative gaze (Foucault, 1977). Other people's comments about their body and general discussions about the body can both lead to young people monitoring their own behavior based on the content and direction of these conversations (Shilling, 2003; Cole et al., 2004; Markula and Pringle, 2007; Hultqvist, 2011). It is also conceivable that this monitoring takes place in a situation of not knowing what people around the individual are thinking, which may give the individual a sensation of not knowing if they are being observed. In connection with the girls' negative experiences of talking about the body in their social environments, one can draw on the omnioptic view, where the many see the many, to also apply to the many *hearing* the many (Jurgenson, 2011). The examples in this study include concrete descriptions of body ideals, discussion of the body in general, and negative comments related to one's own and/or others' bodies. Since young people not only hear such dialogue, but also actively participate in it, they can be described as *prosumers* and *body monitoring* can be viewed as a mutual process (Jurgenson, 2011).

## Summary and Conclusion

In this study, we posed the research question: “How do a select group of third-year upper secondary school students understand body images and body pressures through social media?” Using Foucault's panopticon, we have discovered that young people seem to be subject to various strategic power strategies, illustrated in the BOPS model that we developed.

We perceive the BOPS model as a new and original contribution in the field of research. We believe that the model works well as an interpretive framework in the understanding and application of Foucault's panoptic power mechanism in relation to today's medial body pressure on young people. In the findings, it appears that young people's discourse surrounding the body is influenced by, and created by, both synoptic and omnioptic media power relations. The mass media, the individual, family, friends, and other role models are all identified as relevant actors. A major finding is that it may indicate that the informants are aware of, and do not appear to be merely passive recipients of, a one-side, vertical, medial body pressure, but they themselves are active on social media and thus contribute to the idea of what characterizes a normal or acceptable body.

The strength of the study is that we have researched in a field that seems little explored. Several quantitative studies have been carried out in the field, but fewer strength qualitative studies, especially in a Norwegian context, where the individual youth's “voice” has been heard and interpreted. One of the advantages with individual interviews is that we can explore matters that specifically relate to the informant's subjective opinion. It can also be used to understand connections between the individual informants (Tjora, 2017). Nevertheless, it is conceivable that the power dynamic between informant and researcher can seem intimidating and thus lead to one not being able to obtain all the available information. It is difficult for another researcher to replicate the results obtained by semi-structured interviews as they are a form of social interaction (Jørgensen and Phillips, 1999), and the interpersonal relationship between the researcher and informant, and therefore the answers the informant provides, cannot be replicated (Thagaard, 2018).

There are some more limitations to the present study that should be noted. First, there is a limited selection of informants in the study. Secondly, it is also possible that some informants have failed to answer, or failed to answer in more detail, some of the questions in the interview as the topic is sensitive. And finally, if we had sharpened the topic even more to just body pressure from various social media, we would have been able to make an even more thorough analysis of various aspects of the media's body impact on youth.

In light of the fact that we experience an explosive use of media, not least from today's youth, we will in the future need more qualitative studies that look more closely at various aspects of media related to youth and body image, studies that use both interviews and analyzes of online sources.

## Data Availability Statement

The original contributions presented in the study are included in the article/supplementary material, further inquiries can be directed to the corresponding author.

## Ethics Statement

The studies involving human participants were reviewed and approved by Norsk senter for forskningsdata (NSD). The patients/participants provided their written informed consent to participate in this study. Written informed consent was obtained from the individual(s) for the publication of any potentially identifiable images or data included in this article.

## Author Contributions

All authors have contributed to the article's design and methods, theory, introduction, findings, discussion, and conclusion. IS has also conducted the interviews and systematized the data and OS has been the contact person for the journal and led the revision of the article. All authors have been involved in the entire process of the article and approved the submitted version.

## Conflict of Interest

The authors declare that the research was conducted in the absence of any commercial or financial relationships that could be construed as a potential conflict of interest.

## Publisher's Note

All claims expressed in this article are solely those of the authors and do not necessarily represent those of their affiliated organizations, or those of the publisher, the editors and the reviewers. Any product that may be evaluated in this article, or claim that may be made by its manufacturer, is not guaranteed or endorsed by the publisher.

## Footnotes

^1^Own translations into English of Norwegian names.

^2^Øgård-Repål et al. (2017) undertook focus group interviews with girls in the ninth grade. Østvold (2017) conducted focus group interviews consisting of three groups: six boys and seven girls from the same upper secondary school and four girls from another upper secondary school. He concludes that the pupils were influenced by social media body pressure.

^3^*Ungdata* conduct local surveys in which pupils in lower and upper secondary schools throughout Norway contribute answers to a larger survey, thereby providing a national picture for the period 2017-2019.

^4^They conducted a systematic review of 20 articles, focusing on the effect of social media use on body image and eating disorders.

^5^From the Greek “pan,” meaning “all,” and “optikos,” meaning “sight.” For a closer examination of this model, see https://en.wikipedia.org/wiki/Panopticon.

^6^The Greek prefix “syn” means “with” or “together.”

^7^The Greek prefix “omni” means “everything” and refers to a horizontal, bilateral effect which goes both ways.
